# Supplementary material for: The Quality of Menu Offerings in Independently Owned Restaurants in Baltimore, Maryland: Results from Mixed-Methods Formative Research for the FRESH Trial
Source: Nutrients. 2024 May 18;16(10):1524. doi: 10.3390/nu16101524 (PMC11123881; doi:10.3390/nu16101524)
Supplement: Supplementary file 1 [file nutrients-16-01524-s001.zip › Supplementary Material S1.pdf]

FRESH

FAQ SHEET FOR RESTAURANT OWNERS

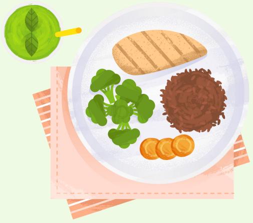

Who are we?

We're a passionate team of public health advocates from Johns Hopkins University and George Washington University. Our overall mission is to foster healthier communities through improved food systems. We have an extensive history of working with various food outlets in the community, including corner stores, dollar stores, grocery stores, carryouts, food pantries, and recreation centers.

What is FRESH?

FRESH is an acronym for *Focus on Restaurant Engagement to Strengthen Health*. It's an initiative to enhance healthy food choices in small, independently-owned carryout restaurants, especially those in urban, underserved neighborhoods. We hope to achieve this by working with restaurant owners (like you!) to refine menu offerings, enhance food preparation techniques, and introduce effective promotion strategies for healthier choices.

Who are we looking to work with?

We are seeking **independently-owned carryout restaurant owners** in **Baltimore and the Washington DC metropolitan areas**, including Prince George's County, Montgomery County, and Washington DC.

What is the ask?

If you are interested in taking part in this exciting project, we will ask for a 45-60 minute interview and an observation of your restaurant operations at your convenience. Furthermore, we may invite you to join a community workshop nearby with other restaurant owners and managers. This workshop will serve as a platform to discuss your experiences, challenges, and victories of running a restaurant in these communities, and help us figure out how we can lend a hand.

What will you gain through participating?

By participating in FRESH, you're showing that you care about the health and wellbeing of your customers and community. In appreciation of your time and involvement, we'll provide a **$40** gift card for participating in the interview.

What will it cost to participate?

There will be no cost to you. Our research team will travel to your restaurant at a time that works for you.

How can you get started?

If interested, please get in touch via the email address below (or reach out on social media!). We'll provide additional information and ensure you are eligible to participate before scheduling an interview. Your involvement is vital to us in shaping healthier food access and choices in our communities.

For more information,
please email Veronica Velez-Burgess at

vvelezb1@jh.edu

And be sure to follow us for updates:

Facebook: @B'more Healthy Food Systems
Instagram: @bmorehealthyfoodsystems
Website: https://healthyfoodsystem.net

FRESH
Focus on Restaurant Engagement to Strengthen Health
