# Supplementary material for: The Quality of Menu Offerings in Independently Owned Restaurants in Baltimore, Maryland: Results from Mixed-Methods Formative Research for the FRESH Trial
Source: Nutrients. 2024 May 18;16(10):1524. doi: 10.3390/nu16101524 (PMC11123881; doi:10.3390/nu16101524)
Supplement: Supplementary file 1 [file nutrients-16-01524-s001.zip › Supplementary Material S2.pdf]

Respondent ID: _________________

FRESH

IN-DEPTH INTERVIEW WITH RESTAURANT OWNERS AND MANAGERS

PI Name: Joel Gittelsohn, Uriyoan Colon Ramos

Study Title: Systems Science Approaches to Improve Access to Healthier Foods: The FRESH Trial (Intervention Trial)

IRB No.: 00024491

PI Version No. / Date: **6**/25/2023

Interviewer Name: ________________________________

Restaurant Name: __________________________________

Date: ____/____/____ Interview start time: ___:___AM/PM

MM DD YY

Role in Restaurant: ______________________________

ADD FRESH FAQ and mission.

We are really interested in understand how to best engage restaurants like yours in promoting healthful food items that you already prepare. Can you assist us with this perspective? etc.. I am especially interested in your thoughts about offering healthy foods on your menu, your views of some possible options to do this, and things that would be helpful or challenging when doing so.

[Consent form here]

A. <u>General background of the restaurant:</u>

1. Tell me about yourself. (where are you from/where did you grow up? What took you to opening a restaurant, etc.) your restaurant. (Probe on the history of the restaurant – when opened? How did they choose location?)
    2. Tell me about your customers. (Probe on how customers have changed over time, what kind of relationship staff have with customers, etc.etc.)
    a. What was your previous history/experience in the restaurant business?
3. Tell me about the kind of food you serve. Probes: why did you choose that for this location? (Probe on how these decisions are made, role of cost, which foods are more profitable, etc.)
4. Where do you get the foods/ingredients/beverages that you sell/serve? (Probe on the distributors, suppliers, etc.) (Try to get history here about various food suppliers, and challenges, facilitators, what they like, disliked about each or this process). How do you keep track of what you need to order and when? (is there an automated system or hand written, etc.
5. How do food/ingredients get transported to the restaurant?
6. Tell me about the types of employees you have at the restaurant. (What are their roles?)
7. Tell me about the methods you use to prepare your food. (Probe on preparation, storage, cooking methods used, etc.) - how are the most popular items prepared/how is x food group prepared

B. Questions based on menu review/observation:

8. Thinking about the foods you offer – what do you think is customers favorite/popular? How are they prepared? Why do you think they like them? Lets look at your menu (Show them a copy/photo of their menu, or look at their actual posted menu board)
    a. Go through items (or groups of items).. how are these prepared? Probe on how much of the food is purchased fresh and prepared on site vs. purchased prepared and just re-heated or served (e.g. prepared sauces, frozen chicken, frozen French fries)
    b. Probe on food cost… how profitable is each item? Is it a big money maker?
9. How often do you change your menu or rotate items on and off? What is your process when you are introducing a new item or changing the menu?
10. Moving forward, are there ways that you would like to change your menu? Adding new items? Items you'd like to remove?
11. What are challenges involved when you add or remove items to your menu? We have observed that your menu … (do the following that are true only)
    a. (Example) …Does not have kids meals. What would you think of having a healthy kids meal?

C. Customers' interest in healthier foods/program approaches:

We want to get your opinion on various strategies that we wish we could try out in restaurant settings in order to improve health and nutrition of customers, and so we are going to ask you the following questions:

14. Looking at your current menu, are there items on it that you would consider more healthy? If yes, why do you consider these healthy? If no, why not? What makes these unhealthy?
15. What do you consider to be healthy or important for a healthy diet?
16. Do you think healthy foods are something that your customers are looking for when they come to your restaurant? Why or why not?
17. What are some examples of new healthier menu choices (entrees, side dishes) you could offer to your customers?
18. How could you incorporate more vegetables (not potatoes, plantains, or other starchy vegetables) into your existing menu?

What do you think about some of these specific changes in your restaurant's menu and cooking practices:

19. Offering vegetables (like cooked greens, grilled vegetables, side salad) as a side dish instead of French Fries?
20. Offering a whole grain such as ________________________? (Provide an example of a whole grain that would be relevant to that restaurant. For example, if the restaurant serves sandwiches, suggest whole wheat bread. If the restaurant offers white rice, suggest brown rice.)
21. What about offering additional beverage choices such as ___________________? (Tailor to the

restaurant. Ask about diet soda and flavored water if these aren’t already offered. Ask about reducing portion sizes if they offer beverages that aren’t pre-packaged such as horchata.)

22. What do you think of using canola oil as your primary cooking oil (not for deep fried foods)?
23. What do you think of using less sugar when preparing foods (not for desserts)?
24. How do you feel about reducing the amount of salt you use and adding other seasonings to enhance flavors? (e.g., swapping half of the salt you use for a salt substitute such as Dash?)
25. If more healthier foods/beverages were provided at your restaurant, do you think your customer’s would eat them? (Please name some healthier foods that you think your customers would eat, that would fit within your current menu.)
26. How do you think customers would respond to healthier cooking methods (e.g., e. substituting healthier fats for butter and lard; replacing some of the salt with other seasonings to enhance flavor.)?
27. How do you think customers would respond to reducing the portion sizes of some items, such as fried chicken or fish, if the amount of foods remains the same by adding healthier sides, such as steamed vegetables or fruit?
28. How do you think customers would respond to reducing portion sizes of some less healthy items? What if the overall amount of food remains the same by adding other healthier ingredients like sides of fruit or vegetables?
29. How do you think your customers would respond to discounts on healthier food and beverages? Is that something you think would boost sales of those items?
30. How do you think your customers would respond to advertisements of healthy foods? How do you think your customers would respond to labeling your menu (added sugar, sodium, calories, and saturated fat) content of your foods?

<u>General Perceptions about FRESH</u>

Now I'd like to ask some questions about your general perceptions of these possible changes. Assu ming you agree with some of the changes, …

32. How do you think that a program like this would be good or bad for your restaurant or the surrounding community? (Probe)
33. What factors do you think might be barriers to involving your restaurant staff in the program?
34. What do you suggest we do to overcome these barriers? (e.g., training)
35. How can we motivate restaurant staff to participate?
36. Would you be willing to advertise healthy foods in your restaurant, if we cover the costs? What kinds of advertising do you find to be most effective?
37. Would you be willing to change your menu to include nutrition information, if we cover the costs?
38. What does success look like
